# A historical case study of early neurosurgery in Ireland: posterior fossa decompression for a skull base tumour in 1922

**DOI:** 10.1007/s11845-026-04491-z

**Published:** 2026-06-15

**Authors:** Ashley Wei, John Caird, Peter Widdess-Walsh

**Affiliations:** 1 School of Medicine, Royal College of Surgeons in Ireland, Dublin, Ireland; 2 Department of Neurosurgery, Beaumont Hospital, Dublin, Ireland; 3 Department of Neurology, Beaumont Hospital, Dublin, Ireland

**Keywords:** Adams McConnell, Brain tumour, Ireland, Neurosurgery history, Ventriculography

## Abstract

The 1920s was a formative period in the development of neurosurgery in Ireland. This study examines a 1922 brain tumour case treated by Adams McConnell, father of Irish neurosurgery, that illustrates the clinical limitations and defining practices of early neurosurgical interventions. A 19-year-old female was admitted with progressive neurological symptoms, including paraesthesias, diplopia, worsening vision, persistent vomiting, and gait imbalance. On clinical examination, signs of severe raised intracranial pressure and brainstem involvement were present, including papilledema and ataxia. Following diagnostic ventriculography, the patient then underwent two surgeries, including attempts at posterior decompression and posterior fossa exploration. Her condition rapidly declined postoperatively, marked by escalating fever and tachycardia, resulting in her death. Autopsy revealed a right-sided skull base tumour originating from the Gasserian ganglion. The tumour had eroded the middle cranial fossa and extended posteriorly to compress the pons, so it was not visible to the surgeons during craniotomy. Without modern medical technology and imaging, careful history taking, meticulous clinical observations, and the neurological examination were relied on to formulate a diagnosis. This study offers a snapshot of early neurosurgery amid diagnostic constraints; through which we can appreciate the remarkable advancements in the field over the past century.

## Introduction: neurosurgery in the early twentieth century

The 1920 s marked a formative period in the development of neurosurgery in Ireland. Adams McConnell, a pivotal figure in Irish neurosurgery, adopted and refined surgical and diagnostic techniques influenced by leading American neurosurgeons Walter Dandy and Harvey Cushing [1, 2].

**Fig. 1:**
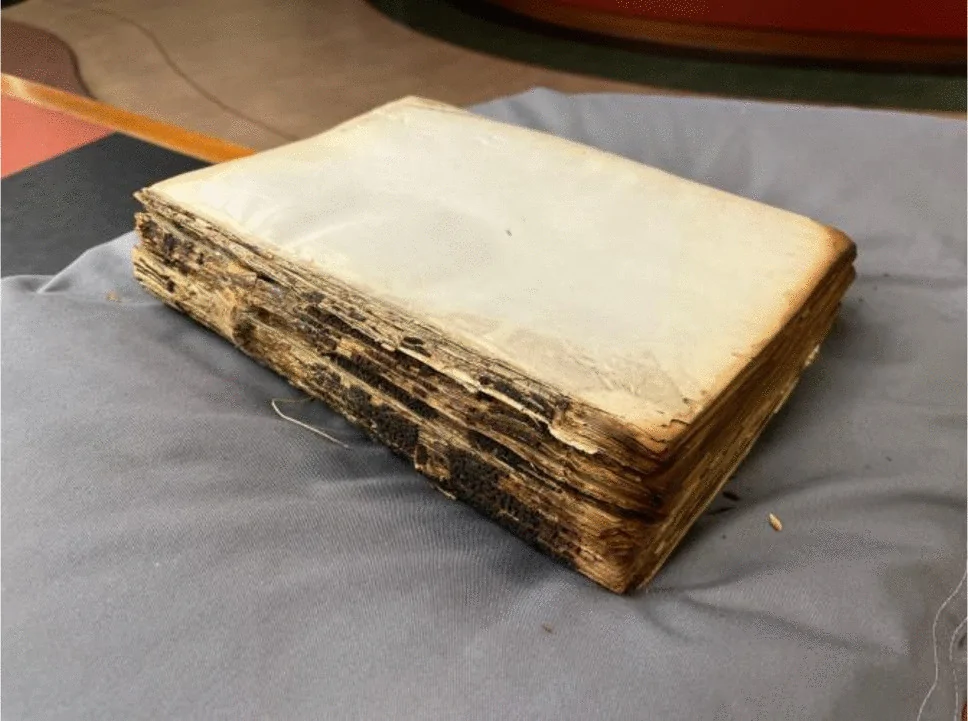
Adams McConnell’s 1922 Casebook, National Archives of Ireland (Record Code: NAI/PRIV 1267/2/120). Photograph by the author, 2024, Bishop Street, Dublin

This study presents a historical analysis of a neurosurgical case under Adams McConnell from 1922 detailed in the Richmond Hospital casebooks located in the Irish National Archives (Fig. 1). The case is reconstructed from contemporaneous casebook descriptions and operative summaries. As is typical of archival clinical records, documentation is incomplete in places; operative positioning, craniectomy dimensions, and detailed intraoperative findings are not consistently recorded. Interpretive comments are therefore distinguished from what is explicitly documented in the primary source. The Richmond Surgical Hospital opened in 1811 and was Ireland’s primary neurosurgical centre until its closure in 1987 [3]. We describe the case of a 19-year-old female with a complex brain tumour, managed by Adams McConnell at the Richmond Hospital (now Beaumont Hospital) in Dublin, Ireland [3]. By examining her detailed clinical course, and the post-mortem findings, this report aims to illustrate the diagnostic and therapeutic challenges, and the operative practices that defined early 20th-century neurosurgery.

## Case presentation and initial diagnostic challenges

A 19-year-old female was admitted to Hamilton ward, Richmond Hospital, on August 25, 1922. Her presenting symptoms had evolved over two months and included:

- A feeling of numbness on the top of her head.
- Persistent double vision (diplopia) and gradually worsening sight, leading to significantly reduced visual acuity (Right eye 6/18, Left eye 6/12).
- Continuous vomiting for two months, occurring day or night, often in the morning, and irrespective of meals. She frequently felt sick before vomiting.
- Unsteadiness in her walk, characterized by staggering and a tendency to fall to the left side (gait ataxic).
- A dull, continuous pain on the top of her head in the middle line was present for four weeks prior to admission.
- Becoming giddy upon stooping.
- Worsening hearing.

Upon examination by the admitting house officer, the patient exhibited significant neurological signs documented in the record:

- Eyes: Pupils were dilated but equal and reactive to light. A well-marked spontaneous nystagmus was observed in both eyes, with diplopia present. Blurred disc outlines and congested vessels were noted, consistent with papilledema and raised intracranial pressure (ICP).
- Reflexes: Knee jerks were absent on both sides, though later notes indicated they were active. Babinski sign was present on the left. No ankle clonus or Kernig sign. Corneal reflex was absent on the left side.
- Coordination: Lack of coordination was evident when attempting to bring both forefingers together and when trying to touch the tip of her nose with eyes closed (finger-nose dysmetria).
- Gait: Ataxic gait with a tendency to fall to the left side.
- Cranial Nerves: Enlarged tonsils, especially on the right. Later observation on September 16th after the first operation suggested facial paralysis, with eyes tightly closing but jaw deviating to the right on mouth closure. Weakness of the left masticatory muscles (pterygoids) was noted, though no paresis of other facial expression muscles [4].

The clinical presentation of vomiting, diplopia, and ataxia strongly suggested a posterior fossa lesion or mass effect. Reduced vision, vomiting, and papilledema were highly concerning for elevated intracranial pressure. While falling to one side is not highly lateralizing, the left Babinski response suggested right-sided lateralization. In combination with an absent left corneal reflex, the signs collectively pointed towards brainstem involvement, particularly the pons. Given the patient’s age and the gradual evolution of symptoms over weeks, a stroke was considered unlikely.

The later observed weakness of the left masticatory muscles (innervated by Cranial Nerve V), facial paralysis, (innervated by CN VII) further supported rising pressure on the brainstem and/or involvement of a skull base process.

### Investigations and surgical interventions

In 1922, without modern neuroimaging, diagnosis often relied on clinical acumen, neurological examination, investigations such as ventriculography and direct exploration.

### Ventriculography (September 5, 1922)

As a diagnostic step, the patient underwent ventriculography, a technique that was considered advanced for its time. Ventriculography was typically done to inform about the size and location of tumor or mass lesion [5]. This patient’s procedure involved passing of a needle into the lateral ventricle under local anesthesia (LA). Fluid began to emerge when the needle had penetrated 1.25 inches from the dura. Seven cubic centimeters (cc) of cerebrospinal fluid (CSF) were collected, and 5 cc of air were slowly injected. After air injection, CSF flow ceased, and no further fluid could be drawn, despite altering the needle direction [4].

This technique, involving the injection of air to outline the ventricles on X-rays, was one of the few ways to evaluate intracranial contents before the advent of CT scanning in 1977 [6, 7]. The procedure described is consistent with ventriculography, where the needle enters at Kocher’s point along the frontal coronal suture line. It is purely diagnostic and not intended to relieve pressure; fluid withdrawal before air injection was crucial to avoid worsening intracranial pressure [5]. The relatively short penetration depth of 1.25 inches suggested a thin cortical mantle, a sign consistent with chronic hydrocephalus.

### First operation: posterior decompression (September 8, 1922)

Following ventriculography, the patient underwent surgery on September 8th, under rectal ether anesthesia (6gm of 50% ether). An incision was made, with the longitudinal part at the external occipital protuberance. Skin, fascia, and muscles were divided to expose the cerebellar bone. The precise configuration and inferior extent of the incision are not specified in the surviving record. A hole was drilled, and bone removed with forceps to enlarge the opening. The notes mention “stitching commenced” and "stitching completed," and "subcutaneous saline given and rectum washed out." There is an unclear note about "pituitary," which might have been pituitary extract for a steroid effect [4].

This approach, involving posterior decompression, suggests that surgeons suspected a mass lesion in the posterior fossa causing brainstem signs and elevated ICP. This technique of removing the posterior bone to reduce pressure is still utilized today. The fact that the patient remained alive for several more weeks post-operatively may be attributed partially to this decompression.

### First post-operative course (September 8 - October 20, 1922)

Initially, the patient developed a fever of 100 °F on September 10th with tachycardia up to 120 beats per minute (bpm). By September 15th, she was afebrile with a heart rate of 70 bpm. Between September 20th and October 5th, blood pressure recordings ranged from 80 to 104. A fever of 99.8°F was noted on September 24th, returning to normal by September 25th. On September 16th, appearance of facial paralysis was noted. While her eyes could be tightly closed, the jaw deviated to the right side upon mouth closure. There was no further paresis of facial expression muscles, but weakness of the left masticatory muscles appeared present [4].

This observed weakness of the left masticatory muscles was likely a feature of rising pressure on the brainstem and, or involvement of a skull base process.

### Second operation (October 20, 1922)

**Fig. 2:**
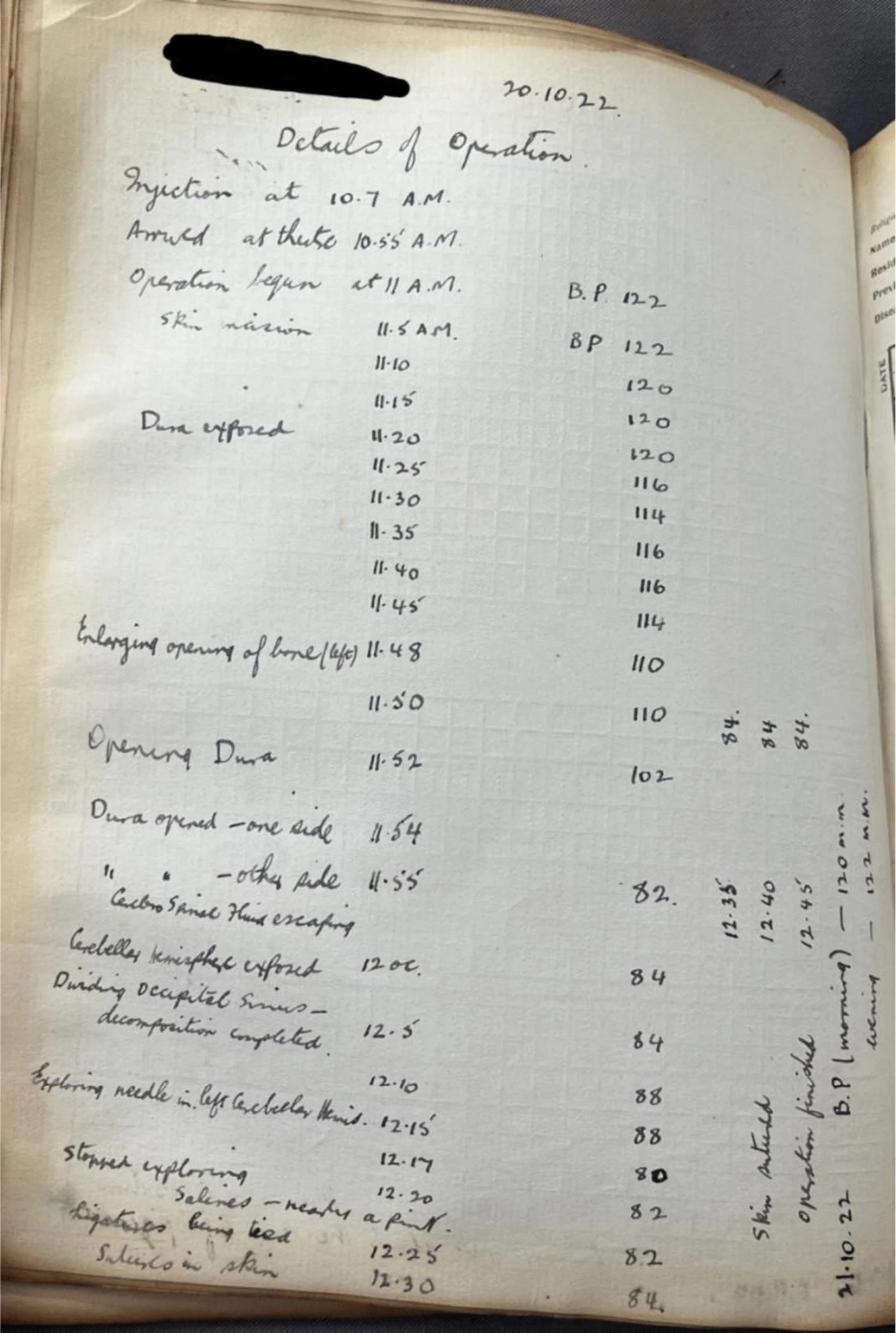
Operative notes dated October 20, 1922, Case No. 33, Adams McConnell Casebook, National Archives of Ireland (Record Code: NAI/PRIV 1267/2/120). Photograph by the author, 2024, Bishop Street, Dublin

The patient underwent a second operation on October 20th, again under rectal ether. A horizontal incision was made through the skin from mastoid to mastoid, slightly upwards, with a vertical incision made downwards in the midline from the horizontal incision. Skin was reflected, and pieces of bone were removed to enlarge the opening. The dura was exposed, opened, and reflected, leading to CSF escape. An “exploring needle” (*likely a Dandy cannula*) was inserted into the left cerebellar lobe but was discontinued as blood pressure began to fall. The wound was closed, and a plaster of Paris cast was applied around the neck, thorax, and head "to steady head." The operation began at 11:00 AM, with skin incision recorded at 11:05 AM, and was completed at 12:45 PM. During the procedure, the patient’s blood pressure fell from 116 to 84 following dural opening (Fig. 2) [4].

This likely represented a further attempt to relieve pressure or identify the underlying pathology through surgical exploration. Without imaging, surgeons of this era often had to surgically explore to understand the intracranial contents. The post-mortem findings (discussed below) later revealed that the tumor was located anteriorly in the middle cranial fossa, extending backward into the posterior fossa. Therefore, the posterior approach adopted in this second operation, targeting the cerebellum, would not have directly visualized the main tumor mass. Post-operatively, the patient showed signs of possible focal seizure (right hand twitching) and left-hand pyramidal weakness with dysarthria [4], potentially indicating right brainstem involvement.

### Clinical course and outcome

Post-operatively after the second surgery, the patient’s condition rapidly deteriorated. On the evening of October 21 st, her BP was 122, but she developed a fever of 103 °F, heart rate of 146 bpm, and respiratory rate of 22. Her fever escalated to 105.6°F on October 22nd, with her heart rate rising to 168 bpm.

The patient died on October 22, 1922, less than 48 hours after her second operation. This rapid decompensation, characterized by rising fever and tachycardia, was consistent with either progressive brainstem compression (including herniation) or postoperative infection (e.g., meningitis/sepsis) [4].

### Post-mortem findings and definitive diagnosis

The post-mortem examination provided the definitive diagnosis and revealed the true extent of the pathology:

- The skull cap was removed, and nothing abnormal was observed on the surface of the brain.
- Upon removal of the brain, a subdural tumor on the right side involving the Gasserian (trigeminal) ganglion was discovered.
- The main tumor mass was approximately the size of a pigeon’s egg (3–4 cm).
- A “diverticulum” (an extension) of the tumor, about half the size of a hen’s egg (5–6 cm), extended posteriorly into the posterior cranial fossa.
- This diverticulum was not adherent to any structures except for its neck, which joined the Gasserian ganglion.
- The tumor had eroded the bone and created a "bed for itself" in the middle cranial fossa.
- Crucially, the tumor had formed a distinct groove in the pons, pushing the latter towards the left.
- The posterior fossa showed no signs of a recent hemorrhage [4].

Based on the post-mortem findings, the diagnosis was most likely a right-sided, slow-growing skull base tumor, possibly a meningioma or a neuroma of the Gasserian (trigeminal) ganglion, with significant extension into the posterior fossa. This mass compressed the brainstem (pons) and caused acute on chronic hydrocephalus.

## Historical analysis and discussion

This case allows a comparison of modern practices to 1922.The patient’s initial presentation (vomiting, diplopia, ataxia, papilledema, cranial nerve palsies) pointed to elevated ICP and posterior fossa involvement. However, without precise diagnostic tools, the exact nature and extent of the skull base tumour remained unclear. In this case, ventriculography yielded limited anatomical clarification. McConnell’s 1921 ventriculography report described replacement of 22–40 cc of CSF with air in published cases [5]. In the present case, only 7 cc were withdrawn and 5 cc injected. The reason for this smaller volume is not specified in the surviving record, but it may have influenced the limited radiographic findings.

This contrasts with modern neuroimaging that allows ready identification of mass lesions. (e.g., MRI, CT).

Antibiotics such as Penicillin were not discovered until 1928 by Alexander Fleming and took time to implement as common practice [8]. Due to the absence of modern intensive care, precise fluid and electrolyte management, or effective antibiotics, complications like severe sepsis or irreversible brainstem herniation due to uncontrolled ICP were often fatal. The high heart rate and fever point to a cascade of events that could stem from medullary compression (due to herniation) or systemic infection, common in pre-antibiotic medicine.

The report is limited in that the archival material does not document ventricular configuration at autopsy, the precise extent of craniectomy, patient positioning, or detailed cortical appearance following dural opening. No histological examination of the tumour was recorded. Consequently, the precise mechanism of death — whether progressive brainstem compression, postoperative infection, or systemic complication — cannot be definitively determined from the surviving notes.

## Conclusion

This 1922 case provides insight into early neurosurgical practice in Ireland, highlighting the diagnostic constraints and operative challenges faced prior to the advent of modern neuroimaging, antibiotics, and critical care. Reconstruction from archival casebooks reveals the uncertainties inherent in clinical decision-making during this formative period. The evolution of imaging and perioperative medicine has since transformed neurosurgical diagnosis and management.

## Data Availability

The archival material supporting this study is cited in the reference list and held by the National Archives of Ireland.

## References

[1] Duddy JC, Kennerk B (1884) Adams Andrew McConnell (1884-1972): pioneer of Irish neurosurgery. Br J Neurosurg 29(1):4–8. 10.3109/02688697.2014.994472 30952182

[2] Andrews H (2009) McConnell, Adams Andrew. In: Quinn J, editor. Dictionary of Irish Biography [Internet]. Royal Irish Academy. [cited 2025 Oct 26]. Available from: https://www.dib.ie/biography/mcconnell-adams-andrew-a5606

[3] O’Brien E, Browne L, O’Malley K (1987) The house of industry hospitals, 1772–1987: the Richmond, Whitworth and Hardwicke (St Laurence’s Hospital): a closing memoir. House of Industry Hospitals, Dublin

[4] McConnell AA (1922) Richmond, Whitworth and Hardwicke Hospitals: Clinical charts (Mr McConnell) [Original record]. Bishop Street, Dublin. Located at: National Archives of Ireland; Record Code: NAI/PRIV 1267/2/120

[5] McConnell AA (1920) Ventriculography. Dublin J Med Sci 2(4):145–151. 10.1007/BF02974823

[6] Pneumoencephalography | MRI, CT & X-Ray | Britannica [Internet]. [cited 2025 Oct 26]. Available from: https://www.britannica.com/science/pneumoencephalography

[7] Schulz RA, Stein JA, Pelc NJ (2021) How CT happened: the early development of medical computed tomography. J Med Imaging Bellingham Wash 8(5):052110. 10.1117/1.JMI.8.5.052110 PMC8555965 34729383

[8] American Chemical Society [Internet]. [cited 2025 Oct 26]. Alexander Fleming Discovery and Development of Penicillin - Landmark. Available from: https://www.acs.org/education/whatischemistry/landmarks/flemingpenicillin.html

